# Nurses’ knowledge, attitudes and practices towards palliative care provided to patients diagnosed with cancer

**DOI:** 10.1371/journal.pone.0289317

**Published:** 2023-10-30

**Authors:** Walaa Mohammed Altarawneh, Rami Masa’deh, Shaher H. Hamaideh, Ahmad M. Saleh, Fadwa Alhalaiqa

**Affiliations:** 1 Oncology Center, Royal Medical Services. Amman, Jordan; 2 School of Nursing, Applied Science Private University, Amman, Jordan; 3 Community and Mental Health Nursing Department. Faculty of Nursing/The Hashemite University, Zarqa, Jordan; 4 Nursing Department, College of Applied Medical Sciences, Prince Sattam Bin Abdulaziz University, Alkharj, Saudi Arabia; 5 College of Nursing, Qatar University, Doha, Qatar; The World Islamic Sciences and Education University, JORDAN

## Abstract

**Aim and objectives:**

This study aimed to explore the levels of knowledge, attitudes, and practices toward palliative care (PC) among nurses working with patients diagnosed with cancer in Jordan.

**Background:**

PC is a growing specialty in healthcare and nursing in Jordan with an increased need to expand its scope, develop policies to govern it, and increase the awareness of its importance especially for patients diagnosed with cancer.

**Design:**

Cross sectional design was used in the current study.

**Methods:**

Using an online self-report questionnaire data was collected from 228 nurses working at four hospitals in Amman. The four hospitals were from three different healthcare sectors: one public, one educational, and two private hospitals. A convenience sampling method was employed.

**Results:**

Results showed a low level of PC knowledge (M = 8.8), a moderate level of attitudes (M = 80.9) toward dying patients, and a moderate level of PC practices (M = 52.7). Differences in PC knowledge, attitudes, and practices were found in regard to nurses’ gender, level of education, PC training, years of experience, and working sector. Significant correlations were found between PC practices with both PC knowledge and attitudes toward dying patients. No significant relationship between PC knowledge and attitudes toward dying patients.

**Conclusion:**

Low level of PC knowledge and moderate level of attitudes toward dying patients. Differences in PC knowledge, attitudes, and practices were found in regard to some participants’ demographics.

## Introduction

Palliative care (PC) is defined by the World Health Organization (WHO) in the Palliative Care Advisory Council in 2018 page 2, as“an approach that improves the quality of life of patients (adults and children) and their families who are facing problems associated with life-threatening illness. It prevents and relieves suffering through the early identification, correct assessment and treatment of pain and other problems, whether physical, psychosocial or spiritual.” [1]. The purpose of PC is not to cure, but to offer comfort and maintain the highest possible quality of life for as long as life is possible [2]. Palliative care practices have developed rapidly in health care institutions over the last decade due to increase of non-communicable diseases, and are now more readily available to patients in need worldwide [3]. PC service is successfully delivered through the combined effect of healthcare professionals’ knowledge, attitudes, and practices [4, 5]. Nada, Nahed [6] reported adequate knowledge, skills, and positive attitudes toward PC enabled nurses to provide high-quality care for chronically ill patients [6].

Due to the global medical improvement in the last decades, the number of people over the age of 65 is rising and life expectancy of those with life-threatening illnesses such as cancer, heart failure, end-stage renal disease, and multiple sclerosis is extended [7]. In Jordan, the aging population is increasing where elder people are living with incurable and life-limiting diseases [8]. More patients require quality PC due to the improved cancer survival rate [9–11]. The PC in Jordan is advanced in comparison to other middle east countries [10] However, it faces many cultural challenges particularly lack of knowledge among healthcare providers and their negative attitudes about pain management, lack of training in opioid and the shortage of trained healthcare professionals [10, 12, 13]. Additionally, lack of education and training programs about PC affect its development and progress negatively [10, 14]. A study conducted by D’emeh, Yacoub [15] found that knowledge deficit and attitudes among nurses regarding pain, particularly in cancer patients, which effect on their ability to assess and treat this symptoms appropriately [15]. Therefore, nurses with insufficient expertise and lack of PC understanding cannot provide adequate abilities to assess patients’ needs, provide appropriate care with high quality [16].

Cancer cases are increasing annually, there were 17 million new cases of cancer worldwide in 2018. Approximately 70% of deaths from cancer occur in low- and middle-income countries [17]. In Jordan, new cancer cases in 2018 were 10898 cases and increased in 2020, which was 11559 cases [18, 19]. PC requires healthcare workers with special competencies to provide continuous and worldwide care associated capacities, commitments, attitudes, knowledge, and practices that enable individuals to behave effectively in their life [7]. Nurses should have adequate knowledge, practice, and a positive attitude to enable them to provide high-quality and effective PC [7]. WHO reported that problems in providing PC might include: the lack of trained human resources, especially health professionals and social workers; a poor understanding of palliative care among health providers; the shortage of hospices and day-care centres; insufficient training for home-based care providers; poor physical health facilities; the lack of a multidisciplinary PC team; inadequate treatment modalities for pain and other symptoms; and an inadequate national palliative care strategy [3]. Healthcare providers play an important role in overcoming these problems.

Numerous studies investigated nurses’ knowledge, attitudes, and practices regarding PC for patients with cancer [4, 20–23]. Morsy, Elfeky [24] conducted a cross-sectional descriptive study in two hospitals in Egypt and determined the nurses’ knowledge and practices of palliative care among cancer patients. Result of this study reported that nurses had poor knowledge and practice of nurses [24]. Moreover, a multicenter cross-sectional Ethiopian study on 422 showed that nurses’ knowledge about PC is inadequate, and showed a negative attitude toward end-of-life care [23].. In addition, a descriptive study among 300 registered nurses revealed poor knowledge toward palliative care while having a favorable attitude toward palliative care [25]. Previous studies reported that nurses’ knowledge and attitudes were correlated with their practices [7, 26]. This explained that nurses who lack knowledge and awareness about PC cannot offer proper practices to evaluate patients’ needs, and so they could not be assigned to PC units [7, 27]. To the researcher’s knowledge, no study in Jordan has assessed nurses’ knowledge, attitudes, and practices about PC. This study aimed to evaluate nurses’ knowledge, attitudes, and practice toward palliative care provided to patients diagnosed with cancer in Jordan. In addition to determine their correlation with participants demographics characteristics and with each other’s.

## Materials and methods

### Design

A cross-sectional, correlational design was used to assess knowledge, attitudes, and practices toward PC among Jordanian nurses. The data was collected in Feb-July 2022. We used the Strengthening the Reporting of Observational Studies in Epidemiology (STROBE) guidelines in reporting current study.

### Setting

The majority of oncology healthcare services are provided within the capital city of Jordan, Amman, it was set to be the target setting for the current study. In order to reasonably increase the variability of the collected data within the targeted city of the study; one main public hospital, two private hospitals, and one educational hospital were included. Adult medical ward, surgical wards, oncology wards, and adult intensive care units within the targeted hospitals were included in the study as patients diagnosed with cancer may get admitted to any of them.

### Sampling and sample size

A convenience sampling method was used to recruit nurses from four hospitals. Power analysis was used to calculate the sample size using the software G*Power^®^ Version 3.1.9.4. Considering a power = 0.8, alpha = 0.05 two-tailed, medium effect size = 0.25; and the number of groups to be compared is five groups using one-way ANOVA test, the minimum sample size needed total sample size of 200 nurses. Additional 10% were added to overcome any missing or incomplete questionnaires.

The inclusion criteria were registered Jordanian nurses having a bachelor’s degree in nursing or higher, have more than one year of clinical experience at wards/units having patients diagnosed with cancer, providing direct bedside patient care at wards/units having patients diagnosed with cancer, took care of dying patients, and able to read, write and understand English (since we provided the tool in English language (however, we did Cronbach alpha to determine the reliability). The exclusion criteria were part-time nurses, and nurses at managerial positions and whose who does not provide direct patient care.

### Instruments

The data collection tool consisted of four sections:

1. *Demographic characteristics includes*: gender, age, years of experience dealing with patients diagnosed with cancer, work setting (Adult oncology Unit, Adult Medical Ward, Adult Surgical Ward, Adult ICU), level of education, hospital type (public, private, educational), and receiving any specialized PC training (Yes/ No).

2. *Palliative Care Knowledge Test (PCKT)*. Knowledge of PC was measured using the 20-item Palliative Care Knowledge Test (PCKT) developed by Nakazawa et al. (2010) [28]. It is a 20 “true and false” questions that test the knowledge of nurses about different domains of the PC. The answers for each question contained the options of “correct = 1”, “incorrect = 2” and “unsure = 3”. Incorrect and unsure answers were coded with 0 and correct answers were coded with 1. The results of the scale were calculated against the correct answers and a total mark (between 0 and 20) was given for each participant. The higher grade indicates a higher knowledge about PC. The same rules of knowledge test scores calculation apply for the scale sub-domains, where the higher the scores (more correct answers) indicate higher knowledge about the domain of the scale being measured. No cut off points were set by the scale’s authors. Moreover, the means of total grades and domains were calculated and compared among the participants’ groups based on their demographics. The domains covered in the scale include: The philosophy (two questions), Pain (six questions), Dyspnoea (four questions), Psychiatric problems (four questions), and Gastrointestinal problems (four questions). The scale was reported to be valid and reliable upon its foundation by Nikazawa et al, 2010 as Cronbach’s alpha was reported to be 0.88 for the overall scale [28]. For this study, the Cronbach’s alpha for PCKT was 0.86.

3. *Frommelt’s Attitude Toward Care of the Dying-form B scale (FATCOD-B)*. The 30-item Frommelt’s Attitude Toward Care of the Dying-form B scale (FATCOD-B) [29] was used to measure attitudes toward PC. The scale consists of 30 statements; each statement scored from 1 (strongly disagree) to 5 (strongly agree). The possible total score ranges from 30 to 150 where the higher score indicate a more positive attitude toward death, as well as toward caring for terminally ill or dying patients. No cut off points were set by the scales authors, so the results were compared to other studies to determine the levels of attitudes toward dying patients among the current study participants.

The scale was translated to many languages and used by many researchers who reported its reliability. The internal consistency was checked, the Cronbach α for FATCOD-B was found to be 0.90 [30]. In Turkish study, Cronbach’s alpha was reported as 0.93 [31]. For this study, the Cronbach’s alpha for FATCOD-B was 0.89.

4. *Palliative Care self-reported Practices Scale (PCPS)*. PC practices were measured by the 18-item Palliative Care self-reported Practices Scale (PCPS) developed by Nakazawa and colleagues (2010) [28]. It includes 18 healthcare practices where the nurse self-evaluated the frequency of doing such a practice with their patients ranging from 1 = not at all, 2 = rarely, 3 = sometimes, 4 = usually, and 5 = always. The possible total score of the scale ranges between 18 and 90, where the higher scores indicate higher frequency of practicing PC. No cut off points were set by the scales authors, so the results were compared to other studies to determine the levels of PC practices among the current study participants. The practice scale measures six nurses practice domains with three questions for each. The domains are: Pain, Dyspnoea, Delirium, Dying-phase care, Communication, and Patient and family centered care. Nakazawa and colleagues (2010) [28] tested the scale which turned to be valid and reliable with a reported Cronbach’s alpha of 0.80 to 0.91 for the whole scale and its subsections. For this study, the Cronbach’s alpha for PCPS was 0.94.

### Data collection procedure

The researcher visited all the adult wards and critical care units at the targeted hospitals and presented to the available staff on duty on each visit about the study purpose, significance, roles and responsibilities of participants, assurance of voluntary participation, and the right to withdraw from the study at any time, and maintenance of confidentiality and privacy of data of the responses. The link for the online questionnaire (Google Forms) was shared with the unit/ward manager and nurses in charge through “whats App” in order to share it with their staff members. The interested participants completed the online self-reported questionnaire until reaching the needed sample size. Therefore, the authors did not have access to information that could identify individual participants during or after data collection.

### Ethical considerations

Ethical approval was obtained from the Institutional Review Board (IRB) of Applied Science Private University (IRB No:2021-2022-2-4).. Also, IRB were obtained from the participating hospitals. Participation in this study was voluntarily. Upon access to online from, an introductory paragraph contains the description of the study purpose and methods and the participant’s rights (to withdraw, maintenance of confidentiality data, and privacy) were confirmed. After reading that, a registered nurse voluntarily decided to participate in the study, the informed consent was obtained by answering the question “I would like to participate in the study” where the participants can click “yes” or “no”. Clicking the “yes” answer is considered as electronic/written informed consent. The informed consent was obtained from all participants for inclusion in the study.

### Data analysis

Statistical Package for the Social Sciences (SPSS) program was used to analyse data. Descriptive statistics (frequencies, means and percentages), independent samples t-test and one-way ANOVA, and Pearson’s Product Moment Correlation analyses were used to meet the research purposes. However, before running all previous inferential statistics, the assumption required for each test were tested and guaranteed. Normal distribution was checked for the main variables and all of them were normally distributed. A p-value of <0.05 was applied to represent the statistical significance of the results, and the level of significance was predetermined as 5%.

## Results

### Sample characteristics

The number of nurses who accessed the questionnaire was 302, 228 completed the questionnaire (response rate = 75.5%). As shown in Table 1, 228 nurses participated in the study, of which 140 were females (61.4%). The average age of participants was 32.5 (SD = 4.7) years ranging between 23 and 42. The average years of experience working with patients diagnosed with cancer was 7.1 (SD = 4.4) with the range between 1 and 18. Nurses from the adult oncology units were 32 (14.0%), 72 (31.6%) from adult medical wards, 64 (28.1%) from adult surgical wards, and 60 (26.3%) from adult intensive care units. Around one third of the participants (n = 36, 15.8%) received PC training, eight of them (3.5%) were having master degree in PC. Other demographic variables were shown in Table 1.

**Table 1:** Participants’ characteristics (N = 228).

Variable	n (%)	Mean ± SD
**Age**		32.5 ±4.7
**Years of experience**		7.1 ±4.4
**Gender**		
Male	88 (38.6)	
Female	140 (61.4)	
**Unit of Work**		
Adult Oncology	32 (14.0)	
Adult Medical	72 (31.6)	
Adult Surgical	64 (28.1)	
Adult Intensive Care	60 (26.3)	
**Hospital type**		
Public Sector	92 (40.4)	
Educational Sector	68 (29.8)	
Private Sector	68 (29.8)	
**Level of Education**		
Bachelor	176 (77.2)	
Graduate (Master/PhD)	52 (22.8)	
**Received Palliative Care Training**		
Yes	36 (15.8)	
No	192 (84.2)	

M: Mean; SD: Standard Deviation

### Levels of PC knowledge, attitudes, and practices

The total mean score of nurses’ knowledge about palliative care is 8.8 (SD = 2.2) which is below that 50% of the test total score. This indicates a low level of PC knowledge among nurses.

Table 2 shows the findings with low knowledge regarding questionnaire subscales Results showed that the mean score of nurses’ attitudes toward dying patient was 80.9 (SD = 32.6) with the minimum total score was 30 and highest total score was 129 on the scale were the possible range is located between 30 and 150.

**Table 2:** Scores of PCKT, FATCOD-B, and PCPS scales and subscales (N = 228).

Scale (Number of items)	Mean ± SD	Lowest Score	Highest Score	Possible Scale Range
**Total PCKT Scale (20)**	**8.8** ± **2.2**	**4**	**14**	**0–20**
Philosophy (2)	0.74 ± 0.71	0	2	0–2
Pain (6)	2.9 ± 0.96	2	5	0–6
Dyspnea (4)	1.74 ± 1.0	0	4	0–4
Psychiatric problems (4)	2.0 ± 0.95	0	4	0–4
Gastrointestinal problems (4)	1.5 ± 0.65	0	2	0–4
**FATCOD-B Scale (30)**	**80.9** ± **32.6**	**30**	**129**	**30–150**
**Total PCPS Scale (18)**	**52.7** ± **19.8**	**19**	**81**	**18–90**
Pain (3)	9.4 ± 4.0	3	15	3–15
Dyspnea (3)	9.0 ± 3.7	3	14	3–15
Delirium (3)	8.4 ± 3.0	3	13	3–15
Dying-Phase care (3)	9.1 ± 3.6	3	15	3–15
Communication (3)	8.9 ± 3.8	3	15	3–15
Patient and Family Centered Care (3)	7.9 ± 2.5	3	12	3–15

**M: Mean; SD: Standard Deviation;** *PCKT*: *Palliative Care Knowledge Test; PCPS*: *Palliative Care self-reported Practices Scale; FATCOD-B*: *Frommelt’s Attitude Toward Care of the Dying-form B scale*

Results showed that the total PCPS mean score of nurses’ palliative care practices was 52.7 (SD = 19.8) in a scale total that ranges between 18 and 90. The lowest total PCPS score was 19 and highest score was 81. At the subscales level see Table 2.

### Differences in levels of PC knowledge, attitudes, and practices according to demographics

In terms of gender, results showed that there was a statistically significant difference (t = -9.33, p<0.001) between male and female nurses in PC knowledge; where males’ mean score (M = 10.3) was higher than females’ (M = 8.0). For attitudes toward dying patients, there was no statistically significant difference (t = 1.25, p = 0.214) between male and female nurses. For PC practices, the results showed that there was a statistically significant difference (t = 5.61, p<0.001) between male and female nurses; where female nurses have higher mean scores (M = 57.9) than males (M = 44.4).

In terms of level of education, the results showed that there was a statistically significant difference (t = -3.9, p<0.001) between bachelor degree holders and nurses with graduate education (Masters and PhD); where nurses with graduate education mean score (M = 9.7) was higher than bachelor degree holders (M = 8.6). For attitudes toward dying patients, there was no statistically significant difference (t = 1.16, p = 0.248) between bachelor degree holders and nurses with graduate education. For palliative care practices, the results showed that there was a statistically significant difference (t = 2.45, p<0.016) between bachelor degree holders and nurses with graduate education; where nurses with bachelor degree have higher mean scores (M = 54.2) than nurses with graduate education (M = 47.8).

In terms of receiving specialized PC training, the results showed that there was no statistical significant difference (t = -3.9, p<0.001) between nurses who received specialized PC training (short course or master degree) and those who did. For attitudes toward dying patients, the results showed that there was a statistical significant difference (t = 3.04, p = 0.004) between nurses who received specialized palliative care training and those who did not; where nurses received the specialized PC training mean score (M = 84.7) was higher than those who did not received the training (M = 64.1). For pPC practices, the results showed that there was a statistical significant difference (t = 5.71, p<0.001) between nurses who received specialized PC training and those who did not; where nurses who received the specialized PC training mean score (M = 55.9) was higher than those who did not received the training (M = 37.5).

In terms of years of experience dealing with patients diagnosed with cancer, there was a statistical significant difference (F = 3.71, p = 0.012) among the nurses with different years of experience in their PC knowledge. In terms of attitudes toward dying patients, the results showed that there was a statistically significant difference (F = 10.05, p<0.001) among the nurses with different years of experience in their attitudes toward dying patients. In terms of PC practices, the results show that there was a statistical significant difference (F = 9.85, p<0.001) among the nurses with different years of experience in their PC practices. Looking into the post hoc testing using Tukey’s test, the result showed that there is a statistically significant difference (p = 0.015) in the knowledge of nurses who have 15 or more years of experience than nurses who have 1–4 years of experience; where nurses with the 15 or more years of experience have higher knowledge mean scores (M = 10.3) than nurses who have 1–4 years of experience (M = 8.4). In terms of attitudes toward dying patients, the nurses with 1–4 years of experience have no statistically significant difference in their attitudes scores (M = 80.0) compared to nurses with 5–9 years of experience (M = 93.4). On the other hand, there was a statistically significant difference among all other comparisons having the nurses with 5–9 years of experience with the highest scores (M = 93.4), nurses with 9–14 years of experience with the score of 75.1 and the lowest scored mean was for nurses with 15 or more years of experience (M = 44.7). For the PC practices mean scores, the nurses with 15 or more years of experience have the lowest (M = 26) and statistically significant different mean scores compared to all the other three groups.

In terms of healthcare sector, the results showed that there was a statistical significant difference (F = 11.2, p<0.001) among the nurses working at different health sectors. In terms of attitudes toward dying patients, the results showed that there was no statistical significant difference (F = 0.318, p = 728) among the nurses working at different health sectors. In terms of PC practices, the results showed that there was a statistical significant difference (F = 5.4, p = 0.005) among the nurses working at different health sectors. Looking into the post hoc testing using Tukey’s test, the result showed that there was no statistically significant difference between private (M = 9.1) and educational (M = 9.6) health sectors in their PC knowledge mean scores. On the other hand, the public health sector did have the lowest knowledge scores (M = 8.1) that is statistically significant compared to private and educational knowledge mean scores. Looking into the PC practices mean scores, the results shows that there is a statistically significant difference only between public and educational health scores in their PC practices; where the public have higher (M = 57.7) means scores than the educational health sector (M = 47.9). (See Table 3).

**Table 3:** Differences in palliative care knowledge, attitudes, and practices based sociodemographic variables.

	Knowledge			Attitudes			Practices		
	M ± SD	F/t	*p*	M ± SD	F/ t	*p*	M ± SD	F/t	*p*
**Gender**		-9.33	**<0.001**		1.25	0.214		5.61	**<0.001**
Male	10.3 ± 2.0			77.5 ± 35.2			44.4 ± 16.1		
Female	8.0 ± 1.7			83.2 ± 30.8			57.9 ± 20.1		
**Level of Education**		-3.9	**<0.001**		1.16	0.248		2.45	**<0.05**
Bachelor	8.6 ± 2.2			82.5 ± 31.4			54.2 ± 20.8		
Graduate	9.7 ± 1.6			76.0 ± 36.1			47.8 ± 15.1		
**Received PC Training**		0.458	0.647		3.04	**0.004**		5.71	**<0.001**
Yes	8.9 ± 2.1			84.7 ± 29.5			55.9 ± 17.7		
No	8.7 ± 2.4			64.1 ± 40.4			37.5 ± 22.2		
**Experience**		3.71	**<0.05**		10.05	**0.000**		9.85	**<0.001**
1–4 years	8.4 ± 2.26			80.8 ± 34.3			56.1 ± 20.4		
5–9 years	8.9 ± 2.57			93.4 ± 30.4			50.1 ± 18.8		
10–14 years	9.1 ± 1.48			75.1 ± 29.4			55.6 ± 17.9		
15 years and more	10.3 ± 0.99			44.7 ± 9.8			26.0 ± 7.4		
**Hospital Name**		11.2	**<0.001**		0.318	0.728		5.4	**<0.001**
Public Sector	8.1 ± 1.78			79.1 ± 32.0			57.7 ± 20.0		
Educational sector	9.6 ± 2.77			83.4 ± 35.1			47.9 ± 20.7		
Private Sector	9.1 ± 1.54			80.8 ± 31.2			50.8 ± 17.1		

* Significant at the 0.05 level (2-tailed), **Significant at the 0.01 level (2-tailed)**M: Mean; SD: Standard Deviation**

### Relationship between PC knowledge, attitudes, and practices

There is a statistically significant positive relationship between nurses’ PC knowledge and palliative care practices (r = 0.350, p<0.001). Also, there is a statistically significant positive relationship between nurses’ attitudes toward dying patient and PC practices (r = 0.600, p<0.000). On the other hand, the results showed no statistically significant relationship between nurses’ PC knowledge and attitudes toward dying patients (r = -0.07, p = 0.3).

## Discussion

### Levels of PC knowledge, attitudes, and practices

The results of the current study showed low levels of knowledge about PC among nurses in Jordan, which is consistent with the findings reported in literature. Ayed and colleagues (2015) [21] shows that about 80% of nurses in Palestine did not have a good level of knowledge about PC. Both settings, here in Jordan and Palestine, may refer this deficiency in knowledge due to the inadequate presence of PC within the curricula of basic nursing education preparation. Other reasons that were discussed also by Ayed and colleagues (2015) [21] were that the nursing staff work overload due to staff shortage which may lead to decreased interest in gaining specialized knowledge about PC, and the absence of specialized PC units within the healthcare institutions. Same results were confirmed in the review of 19 studies conducted by Alshammari, et al., (2022) [32]. They reported that nurses working in non-specialized PC units had lack the knowledge about PC and there was a need to include specialized education for nurses during the undergraduate levels in addition to the educational efforts during clinical practice. As reported in literatures, absence of PC education at the undergraduate levels and insufficient specialized during clinical practice, staff shortage, limited presence of specialized PC units, and limited presence of PC related polices lead to low levels of knowledge about PC among nurses; which in turns has its repercussion on the levels of attitudes toward dying patients and PC practices [21, 32, 33]. However, Getie, Wondmieneh [34] conducted a study among nurses working North Wollo hospitals in Ethiopia. They found that nurses had knowledge 59.7% and 44.2% had a favorable attitude towards PC. Which was higher than the findings of the studies done in Jordan (current study), and Palestine [21]. This could be rationalized by the difference in the study period, settings, and cultures. Additionally, It was also highlighted by the WHO that PC at the developing countries is lacking the needed health policies and strategies that address the main problems in providing PC. The lack of trained health professionals and social workers; a poor understanding of PC among health providers; the shortage of specialized hospice and PC centers, and the inadequate funds for PC related activities; [35] were all among the factors that can be another reason for the low levels of PC knowledge among nurses in Jordan. This findings are similar to the other studies that have been conducted among nurses student and among doctors in which they have in adequate PC’s knowledge [36–38].

The poor nurses’ knowledge about PC was reported to lead to the lack of attitudes toward dying patients and lower PC practices as well [20, 21, 33]. However, at the current study, and despite the low levels of PC knowledge, the result shows moderate levels of attitudes toward dying patients among nurses in Jordan. The results were consistent with what was concluded by Ayed et al. (2015) [21], Kasaa et al (2014) as nurses in Palestine and Ethiopia have moderate levels of positive attitudes toward PC as well. It was referred to cultural background and the comfort levels of nurses in talking about death and dying with patients and families [27]. In another study in Jordan, Al Qadire (2020) [39] reported that nursing students’ attitudes toward dying patients were with an average of 95.8 (SD 8.7) using the same scale of the current study (FATCOD-B). Al Qadire also confirmed the need to include specialized PC education within the undergraduate nursing programs to enhance the attitudes toward dying patients among nursing students.

In terms of practices, the results of the current study also confirmed the moderate levels of PC practices among nurses in Jordan. Khader (2017) [10] directed the focus of explaining conservative PC practices in Jordan was due to the inadequate training and education about PC, and the shortage of trained healthcare professionals is actually a vital barrier that prevents PC development.

### Differences in PC knowledge, attitudes, and practices according to demographics

The results of this study showed that males nurses reported higher levels of PC knowledge, similar levels of attitudes toward dying patients, and lower PC practices than females. Similar levels of attitudes toward dying patients between male and female nurses was consistent with the findings in literature, but it was inconsistent with the reports in literature in terms of the levels of knowledge, or PC practices [27, 40]. The results at the current study can be due to some situational factors that are related to the study sample as male nurses who participated in the study were only 38.6% of the sample only and male nurses with graduate education were 32 males compared to 20 female nurses only. In terms of receiving specialized PC training male and female nurses were equal in numbers of 20 each. Receiving graduate education can be the reason for the higher levels of PC knowledge among male nurses compared to females. Having similar levels of attitudes toward dying patients can be referred to the similar training and education at the undergraduate level of education.

Having lower levels of PC practices among male nurses can be related to the situation where male nurses, since they have higher levels of graduate education, they may tend to be assuming managerial positions like being in charge of the shift duty which may keep them more distant from practicing with patients than bedside nurses. This confirmed by the results of the current study were nurses with graduate education reported higher levels of PC knowledge, similar levels of attitudes toward dying patients, and lower PC practices than nurses with bachelor degree. These results are consistent with the results of Ayed et al. (2015) [21] in Palestine, Kassa et al (2014) [27] in Ethiopia, Bilal (2018) [33] in Sudan. But these results were inconsistent with Paknejadi et al (2019) in Iran, where nurses with higher education did have the same levels of PC knowledge, attitudes toward dying patients and PC practices as the nurses with undergraduate education which can be related to situational factors that are related to the study participants.

The results of the current study show that nurses who received specialized PC training or education in the form of short courses or master degree did actually have higher levels of positive attitudes toward dying patients, and higher levels of PC practices. On the other hand, they have similar levels of knowledge compared to nurses who did not receive specialized PC education. The results were consistent with what was reported by Ayed et al. (2015) [21], Kassa et al (2018) [27] in Ethiopia, Bilal (2018) [33] in Sudan and inconsistent with Paknejadi et al (2019) in Iran. This may direct the attention toward looking into the content of the specialized PC education as it may focus on attitudes and PC practices more that the theoretical PC knowledge.

The thought of effects of specialized PC education courses was also noticed and confirmed by the current study findings where nurses with higher years of experience have higher levels of PC knowledge but lower attitudes toward dying patients and lower PC practices. Specialized PC courses seems to focus mainly on attitudes and PC practices while minimal focus on knowledge can be found as it can be gained through experience in dealing with PC patients. The results related to the years of experience were consistent with Ayed et al. (2015) [21], Bilal (2018) [33], and inconsistent with Paknejadi et al (2019) [40] and Kassa et al (2014) [27].

In terms of working sector, knowledge was the lowest in public sector, similar levels of attitudes toward dying patients, and higher levels of PC Practices compared to private and educational sectors. The results were inconsistent with the reports in literature. Kassa et al. (2014) reported a lower levels of attitudes toward dying patients at nongovernmental hospitals and was referred to the lower and less frequent training received at nongovernmental hospitals compared to governmental ones [27]. While, Bilal (2018) [33] in Sudan reported no difference in knowledge among the working sectors.

### Relationships among PC knowledge, attitudes, and practices

The result show that there is a statistically significant relationship between nurses’ levels of PC practices with the levels of PC knowledge and nurses’ attitudes toward dying patients. This is consistent with the findings in literature where the PC practices is affected by the PC knowledge as well as the attitudes toward dying patients [21, 33]. This finding concurs with Blooms taxonomy (1956) of educational outcomes where he describes that practices are affected by the knowledge and attitudes as prerequisites for putting skills and abilities into executing the needed practices [41].

The current study shows that nurses in Jordan have moderate levels of PC practices which, according to Blooms, can be referred to the similar levels of moderate positive attitudes toward dying patients, but it does not support the other sections of Blooms when looking into the low levels of PC knowledge among nurses in Jordan. This can be a dilemma to be more investigated looking into the situational factors that lead to this results which can be the limited PC continuous education opportunities for nurses at the field; while the higher levels of attitudes with the concurrent higher levels of PC practices were enforced by the general nursing professional and ethical commitments and standards in carding for patient in general and toward patients diagnosed with cancer in specific. The debate of disconnect was shown in the result where there was no statistically significant relationship between nurses’ PC knowledge and attitudes toward dying patients. This is inconsistent with the results reported in literature [21, 33, 27].

Despite the strength of the current study in exploring the Jordanian nurses’ knowledge, attitudes toward dying patients and PC practices at different healthcare sectors in Jordan, it includes some limitations. The descriptive study design, using convenient sampling and the fact that it was conducted at one setting (Amman) may limit the generalizability of the study findings to the different healthcare settings in Jordan, in the region, or globally. Additionally, The lack of specialized PC units at the healthcare institutions in Jordan. The using of using of English version of instruments might also limit the generalizability of the current study findings. Therefore, future studies must take these limitations on consideration. We recommend futures studies to be done with including healthcare professional other than nurses, different settings to enhance the representation of the study and using validated Arabic versions of the instrument. In addition to, interventional programs that could enhance knowledge, attitudes, and practices among nurses.

## Conclusions

Results showed a low level of PC knowledge, a moderate level of attitudes toward dying patients, and a moderate level of PC practices. Males nurses reported higher levels of PC knowledge and lower PC practices than females. Nurses with graduate education reported higher levels of PC knowledge and lower PC practices than nurses with bachelor degree. Nurses who received PC training reported higher levels of attitudes toward dying patient and higher levels of PC practices than nurses who did not receive any specialized PC training. The results also showed that nurses with higher years of experience have higher levels of PC knowledge but lower attitudes toward dying patients, and lower PC practices.

### Relevance to clinical practice

The study’s findings affirmed the importance of inclusion of palliative care management in basic and advanced nursing education programs, which will help future nurses become more skilled and efficient in providing optimal cancer nursing evaluation and care. Furthermore, by utilizing the study data concerning cancer patients in Jordan, it may serve as a foundation for undergraduate and postgraduate students in activating the evidence-based practice of palliative care management incorporated in theoretical and clinical training courses to enhance knowledge attitudes and practice toward PC.

## Supporting information

S1 Checklist (DOCX)

S1 Data (XLSX)

## References

[1] World Health Organization. Noncommunicable diseases 2023 [Available from: https://www.who.int/news-room/fact-sheets/detail/noncommunicable-diseases.

[2] Cha E, Lee S, Lee J. Health Personnel’s Knowledge, Attitudes, and Self-Efficacy Related to Providing Palliative Care in Persons with Chronic Diseases. The Korean Journal of Hospice Palliative Care. 2020;23(4):198–211. doi: 10.14475/kjhpc.2020.23.4.198 37497473 PMC10332728

[3] World Health Organization. Palliative care 2023 [Available from: https://www.who.int/news-room/fact-sheets/detail/palliative-care.

[4] Farmani AH, Mirhafez SR, Kavosi A, Pasha AM, Mohammadi G, Moeini V, et al. Dataset on the nurses’ knowledge, attitude and practice towards palliative care. 2019;22:319–25. 10.1016/j.dib.2018.11.133 PMC6307337 30596126

[5] Gopal KS, Archana PSJIJOSS. Awareness, knowledge and attitude about palliative care, in general, population and health care professionals in tertiary care hospital. 2016;3(10):31–5.

[6] Nada M, Nahed A, A. F. A Scoping Review on Palliative Care: Knowledge and Attitude of Nurses. Open Access Journal of Biomedical Science. 2021;3(2).

[7] Huang L-C, Tung H-J, Lin P-CJPo. Associations among knowledge, attitudes, and practices toward palliative care consultation service in healthcare staffs: A cross-sectional study. 2019;14(10):e0223754. 10.1371/journal.pone.0223754 PMC6788675 31603946

[8] Help Age International Jordan. A Profile of Older People in Jordan: The Experiences & Inclusion Risks of Syrian Refugees & Older Jordanians. 2018.

[9] Park M, Yeom H-A, Yong SJJBPC. Hospice care education needs of nursing home staff in South Korea: a cross-sectional study. 2019;18:1–8. 10.1186/s12904-019-0405-x PMC6373091 30755208

[10] Khader K. Cultural challenges in implementing palliative care services in Jordan. Palliative Medicine Hospic Care Open Journal 2017;1:68–72.

[11] Al-Ghabeesh SH, Al-Kalaldah M, Rayan A, Al-Rifai A, Al-Halaiqa F. Psychological distress and quality of life among Jordanian women diagnosed with breast cancer: The role of trait mindfulness. Eur J Cancer Care (Engl). 2019;28(5):e13082. doi: 10.1111/ecc.13082 31066145

[12] Al Khalaileh M, Al Qadire M. Barriers to cancer pain management: Jordanian nurses’ perspectives. Int J Palliat Nurs. 2012;18(11):535–6, 8–40. doi: 10.12968/ijpn.2012.18.11.535 23413501

[13] Othman EH, Khalaf IA, Alosta MR, Abualruz H, Zeilani R. Death and Dying Through the Lens of Jordanian Muslim Patients and Caregivers. Omega (Westport). 2022:302228221133505. doi: 10.1177/00302228221133505 36223981

[14] Abdalrahim MS, Herzallah MS, Zeilani RS, Alhalaiqa F. Jordanian nurses’ knowledge and attitudes toward cancer-related fatigue as a barrier of fatigue management. J Am Sci. 2014;10(2):191–7.

[15] D’emeh W, Yacoub M, Darawad M, Al-Badawi T, Shahwan B. Pain-Related Knowledge and Barriers among Jordanian Nurses: A National Study. Health. 2016;8:548–58.

[16] Khalaf IA, Al-Dweik G, Abu-Snieneh H, Al-Daken L, Musallam RM, BaniYounis M, et al. Nurses’ Experiences of Grief Following Patient Death: A Qualitative Approach. J Holist Nurs. 2018;36(3):228–40. doi: 10.1177/0898010117720341 28845718

[17] United Nations Population Division [UNPD]. Life expectancy at birth for both sexes combined 2021 [Available from: http://data.un.org/Data.aspx?q=jordan&d=PopDiv&f=variableID:68;crID:400.

[18] Khaled H, Ibrahim A, Khaled R. Cancer incidence and mortality for 22 Arab countries in 2020: Profile of total and top major cancers. Research Square. 2022.

[19] Maabreh RS, Al-Husban RY, Al-Akash HY, Al-Shdayfat N. Women’s health concern in Jordan: knowledge, practice and barriers toward cervical cancer screening. International Journal of Human Rights in Healthcare. 2021;ahead-of-print(ahead-of-print).

[20] Anteneh S, Kassa H, Demeke T, Guadu T. Assessment of nurses’ knowledge, attitude, practice and associated factors towards palliative care: in the case of Amhara region hospitals. Journal Advances in Biological Research. 2016;10(2):110–23.

[21] Ayed A, Sayej S, Harazneh L, Fashafsheh I, Eqtait F. The Nurses’ Knowledge and Attitudes towards the Palliative Care. Journal of Education Practice. 2015;6(4):91–9.

[22] Taber JM, Ellis EM, Reblin M, Ellington L, Ferrer RA. Knowledge of and beliefs about palliative care in a nationally-representative U.S. sample. PLoS One. 2019;14(8):e0219074. doi: 10.1371/journal.pone.0219074 31415570 PMC6695129

[23] Etafa W, Wakuma B, Fetensa G, Tsegaye R, Abdisa E, Oluma A, et al. Nurses’ knowledge about palliative care and attitude towards end- of-life care in public hospitals in Wollega zones: A multicenter cross-sectional study. PLoS One. 2020;15(10):e0238357. doi: 10.1371/journal.pone.0238357 33027265 PMC7540839

[24] Morsy WYM, Elfeky HAA, Mohammed SE. Nurses’ Knowledge and Practices about Palliative Care among Cancer Patient in a University Hospital ‐ Egypt. Advances in Life Science and Technology. 2014;24:100–13.

[25] Parveen A, Sultana K, Waqas, Tasneem S, Jabeen R, Faiz A, et al. Knowledge and attitude of nurses about palliative care. Journal of Bioresource Management. 2020;7(1):8.

[26] Mengual TE, Chover-Sierra E, Ballestar-Tarín ML, Saus-Ortega C, Gea-Caballero V, Colomer-Pérez N, et al. Knowledge about Palliative Care and Attitudes toward Care of the Dying among Primary Care Nurses in Spain. Healthcare (Basel). 2023;11(7). doi: 10.3390/healthcare11071018 37046946 PMC10094341

[27] Kassa H, Murugan R, Zewdu F, Hailu M, Woldeyohannes DJBpc. Assessment of knowledge, attitude and practice and associated factors towards palliative care among nurses working in selected hospitals, Addis Ababa, Ethiopia. BMC Palliat Care. 2014;13(1):1–11. 24593779 10.1186/1472-684X-13-6 PMC3975866

[28] Nakazawa Y, Miyashita M, Morita T, Umeda M, Oyagi Y, Ogasawara T. The palliative care self-reported practices scale and the palliative care difficulties scale: reliability and validity of two scales evaluating self-reported practices and difficulties experienced in palliative care by health professionals. J Palliat Med. 2010;13(4):427–37. doi: 10.1089/jpm.2009.0289 20384502

[29] Frommelt KHMJAJoH, Medicine® P. Attitudes toward care of the terminally ill: an educational intervention. 2003;20(1):13–22. 10.1177/104990910302000108 12568433

[30] Hasheesh MOA, AboZeid SA-S, El-Said SG, Alhujaili ADJHsj. Nurses’ characteristics and their attitudes toward death and caring for dying patients in a public hospital in Jordan. 2013;7(4):384.

[31] Cevik B, Kav S. Attitudes and experiences of nurses toward death and caring for dying patients in Turkey. Jounrnal of Cancer nursing. 2013;36(6):E58–E65. doi: 10.1097/NCC.0b013e318276924c 23151504

[32] Alshammari F, Sim J, Lapkin S, Stephens M. Registered nurses’ knowledge, attitudes and beliefs about end-of-life care in non-specialist palliative care settings: A mixed studies review. Nurse Educ Pract. 2022;59:103294. doi: 10.1016/j.nepr.2022.103294 35078071

[33] Bilal M. The Knowledge of Palliative Care and the Attitude Toward it among the Nurses at Sabia General Hospital 2018. Sudan Journal of Medical Sciences. 2018;13(4):301–10.

[34] Getie A, Wondmieneh A, Mengesha A, Fitwi A, Gedefaw G, Demis A. Assessment of Knowledge and Attitude towards Palliative Care and Associated Factors among Nurses Working in North Wollo Hospitals. Ethiop J Health Sci. 2021;31(2):393–400. doi: 10.4314/ejhs.v31i2.22 34158791 PMC8188070

[35] Carpenter JG, Hanson LC, Hodgson N, Murray A, Hippe DS, Polissar NL, et al. Implementing Primary Palliative Care in Post-acute nursing home care: Protocol for an embedded pilot pragmatic trial. Journal of Contemporary Clinical Trials Communications. 2021;23:100822. doi: 10.1016/j.conctc.2021.100822 34381919 PMC8340123

[36] Gelegjamts D, Yong Yoo J, Kim J, Sun Kim J. Undergraduate nursing students’ palliative care knowledge and attitudes towards end-of-life care: a cross-sectional descriptive study. Contemporary Nurse. 2020;56(5–6):477–90. doi: 10.1080/10376178.2021.1890165 33573520

[37] Ismaile S, Alshehri HH, Househ M. Knowledge of Palliative Care Among Nursing Students. Stud Health Technol Inform. 2017;238:261–4. 28679939

[38] Fadare JO, Obimakinde AM, Afolayan JM, Popoola SO, Aduloju T, Adegun PT. Healthcare workers knowledge and attitude toward palliative care in an emerging tertiary centre in South-west Nigeria. Indian J Palliat Care. 2014;20(1):1–5. doi: 10.4103/0973-1075.125547 24600175 PMC3931235

[39] Al Qadire MJO-JoD Dying. Jordanian student nurses’ attitudes towards the care of dying patients. 2022;86(2):382–94. 10.1177/0030222820971077 33115333

[40] Paknejadi F, Hasavari F, Khaleghdoost Mohammadi T, Kazemnejad Leili E. Nurses’ Knowledge of Palliative Care and Its Related Factors. Journal title. 2019;29(4):236–42.

[41] Armstrong P. Bloom’s Taxonomy. Vanderbilt Center for Teaching, Nashville.. 2010.

